# Evaluation of Short-Term Toxicity and Cholesterol-Lowering Effects in Rats Exposed to Dietary Fiber Derived from Cassava Pulp

**DOI:** 10.3390/foods12224074

**Published:** 2023-11-09

**Authors:** Kakanang Posridee, Pornariya Chirinang, Anant Oonsivilai, Ratchadaporn Oonsivilai

**Affiliations:** 1 School of Food Technology, Institute of Agricultural Technology, Suranaree University of Technology, Nakhon Ratchasima 30000, Thailand; posridee.ka@gmail.com; 2 Applied Food and Nutrition Division, Faculty of Science and Technology, Phetchaburi Rajabhat University, Phetchaburi 76000, Thailand; pornariya.chi@mail.pbru.ac.th; 3 School of Electrical Engineering, Institute of Engineering, Suranaree University of Technology, Nakhon Ratchasima 30000, Thailand; anant@sut.ac.th

**Keywords:** hypocholesterolemia, short-term toxicity, neutral detergent fiber, cassava pulp

## Abstract

The dietary fiber extracted from cassava pulp, composed of crude fiber, neutral detergent fiber (NDF), and cellulose content, demonstrates promise as a functional food ingredient. The study’s objectives encompassed the assessment of short-term toxicity and the evaluation of its potential cholesterol-lowering effects. The results indicated that cassava pulp dietary fiber (CDF) is well-tolerated with non-toxic thresholds determined at 10.01 g/kg body weight/day for male rats and 11.21 g/kg body weight/day for female rats during the short-term toxicity assessment. Furthermore, CDF exhibited notable cholesterol-lowering effects, significantly reducing serum triglyceride and serum total cholesterol levels, along with decreased liver total lipids and liver cholesterol levels. In contrast, it led to significant increases in fecal total lipids and cholesterol when compared to the control group. Most notably, there were no significant differences in terms of serum triglyceride, serum total cholesterol, liver total lipids, and liver cholesterol between CDF and the conventional cholesterol-lowering medication, simvastatin. These findings underscore the potential of cassava pulp dietary fiber as a natural and safe alternative for managing hyperlipidemia and related conditions. It offers a valuable avenue for the development of functional foods aimed at improving cardiovascular health and further investigation for its potential application in the field of nutraceuticals.

## 1. Introduction

Cassava (*Manihot esculenta Crantz.*) is the third-largest source of food carbohydrates in the tropics. Cassava is mostly grown in tropical countries. Nigeria is the world’s largest cassava producer, followed by Brazil and Thailand. However, Thailand had a higher yield per area than Nigeria and Brazil. Thailand is currently one of the world’s largest exporters of cassava products. Cassava was planted in all regions of Thailand except the South. In year 2015, more than 50% was planted in the northeast, where the main province is Nakhon Ratchasima, followed by the Central Plain (33%) and the North (15%). These planted areas included 48 provinces or approximately 8.8 million raises, and the total production was approximately 33 million tons. Fifty percent of this was used as raw material for the production of tapioca or cassava starch and the other 50 percent as cassava chips, cassava pellets, and cassava flour. Cassava starch production is one of the most important agro-industries in Thailand. Thailand is the third largest producer of cassava starch, which yields 2.9 million tons per year and has a value of approximately 41.2 billion baht. Consequently, there are numerous byproducts from the processing of cassava, of which the most important is cassava pulp. At least 1 million tons of pulp are generated annually in Thailand. Cassava pulp represents approximately 10–15% by weight of the original cassava roots. The fiber content of dried cassava pulp was reported in the form of insoluble fiber. Moreover, the pulp also contains pectin, cellulose, and approximately 10–15% fiber, 1.5–5% protein, and 0.1–4% fat. Historically, it has been used in the animal feed industry, biogas production, and ethanol production [1,2]. Due to its high content of fiber, cassava pulp could be used as a fiber-rich ingredient in food products. Wandee et al. [3] evaluated the potential of cassava pulp and pomelo peel as sources of dietary fiber in dried rice noodles. The results show that a combination of cassava pulp and pomelo peel in rice noodles at a total amount of 20% exhibited an obvious increase in cooking weight, and its highest total dietary fiber content was 14.4%. Thus, a purification method with mild and highly specific conditions with enzymatic hydrolysis to obtain more dietary fiber content from cassava pulp might be very useful. However, there are no published studies on the cholesterol-lowering properties of dietary fiber in cassava pulp. Therefore, the purpose of this study was to determine the physicochemical properties of cassava pulp and to evaluate the potential hypocholesterolemic action of dietary fiber from cassava pulp on rats that were fed a high-fat diet.

Short-term toxicity refers to the adverse effects that occur following oral or dermal administration of a substance that result either from a single exposure or from multiple exposures in a short period of time. Moreover, the adverse effects of toxicity should occur up to 14 days after the administration of the substance in animal models, which are typically mice or rats [4].

Marques et al. [5] conducted a study in hypertensive mice to evaluate the effects of dietary fiber and acetate supplementation on the gut microbiota and the prevention of hypertension and heart failure. In the deoxycorticosterone acetate (DOCA)-salt model, dietary fiber and acetate supplementation caused alterations in the gut microbiota, which inhibited the development of hypertension and associated renal and cardiac fibrosis. Mitamura et al. [6] investigated the effects of water-soluble soybean fiber on ovariectomy-induced osteopenia and hypercholesterolemia in rats. The study found that dietary fiber, particularly viscous dietary fiber, reduced serum cholesterol levels in rats.

Dabour et al. [7] investigated the ability of yogurt supplemented with dietary fibers or bran extracts taken from wheat or rice to lower serum lipids and improve liver function in male hypercholesterolemic rats. The results showed that rats given yogurt supplemented with dietary fibers had lower serum glucose levels. Anderson et al. [8] published a review of the research on the association between dietary fiber and coronary heart disease. The review highlighted various research that found dietary fiber to have cholesterol-lowering effects in both people and rats.

Esmael et al. [9] studied the hypolipidemic effect of fruit fibers in rats on a high-fat diet. The long-term consumption of fruits as a source of dietary antioxidants and fibers was found to be helpful in decreasing total blood cholesterol in rats on a high-fat diet. El-Sayed [10] investigated the effect of dietary fiber feeding on blood cholesterol levels in hypercholesterolemic rats. In hypercholesterolemic rats, supplementing the basal diet with dietary fibers resulted in a significant increase in body weight, body weight gain, food intake, and food efficiency ratio.

In conclusion, research on dietary fiber and hypercholesterolemia in rats from 2018 to 2023 has demonstrated that dietary fiber supplementation can improve serum cholesterol levels and prevent the development of hypertension and heart failure. These findings indicate the potential of dietary fiber as a therapeutic method for controlling cholesterol-related disorders. Cassava pulp is a rich source of dietary fiber, but its short-term toxicity and cholesterol-lowering effects are not well-known. This study aims to comprehensively explore these effects in Wistar rats.

## 2. Materials and Methods

### 2.1. Materials and Sample Preparation

Cassava pulp was collected from Sanguan Wongse Industries Co., Ltd. in Nakhon Ratchasima Province, Thailand. Heat-stable α-amylase Termamyl 120 L (EC 3.2.1.1, Merk, Darmstadt, Germany), amyloglucosidase AMG 300 L from Aspergillus niger (EC 3.2.1.3, Bray, Co., Wicklow, Ireland), and neutrase^®^ (EC 3.4.24.28 from Bacillus amyloliquefaciens, Novozymes Co., Bagsvaerd, Denmark) were used. All chemicals used were reagent grade. A normal rat diet (082G/15, C.P. mice Feed, Perfect Companion Group, Bangkok, Thailand) was used for the control group.

Cassava pulp was dried at 60 °C in a tray dryer (Kluaynumtaitowop, Bangkok, Thailand) for 24 h. Before use, the dried cassava pulp was finely ground (GmbH & Co., KG D-42781, Haan, Germany) and stored at room temperature in a vacuum-packed container.

### 2.2. Dietary Fiber Preparation

Dietary fiber from cassava pulp was prepared, following the method described by [11]. Cassava pulp solution was prepared at 4% (*w*/*v*) with phosphate buffer (50 mM, pH 6). Heat-stable α-amylase Termamyl 0.1% (*w*/*v*) (EC 3.2.1.1, Merk, Darmstadt, Germany) underwent a 30 min treatment at pH 6 and 95 °C and was adjusted to pH 7.5 with 0.17 M sodium hydroxide solution (NaOH) (Merck Ltd., Darmstadt, Germany) prior to adding neutrase (1% *v*/*v*). The neutrase was treated for 30 min at 60 °C and was adjusted to pH 4.5 with 0.205 M phosphoric acid solution (Carlo). Subsequently, 0.1% (*v*/*v*) amyloglucosidase was added to the mixture and treated for 30 min at 95 °C. The resulting hydrolysate was separated via centrifugation (Hettich, Universal 32R, DJB Labcare Ltd., Newport Pagnell, UK) at 10,000× *g* for 10 min to separate the supernatant from the fiber-enriched sediment. The sediment was washed with distilled water, centrifuged again for 10 min at 10,000× *g* and dried at 60 °C in a hot air oven. The dietary fiber powder was kept in a sealed container at 4 °C until use.

### 2.3. Short-Term Toxicity Study of Dietary Fiber

#### 2.3.1. Diet Preparation 

The rat diet was fortified with CDF in a mixed normal rat diet (082G/15, C.P. mice Feed, Perfect Companion Group, Bangkok, Thailand) at 2.5 and 15% (*w*/*w*). The mixed CDF diet was reformed using an extruder (Tricool Engineering Ltd., Hants, UK).

#### 2.3.2. Experimental Protocol

The modified procedure of the short-term toxicity study [12] was carried out. All animals were handled according to Guidelines of the Animal Care and Use of Laboratory Animals from the Center for Laboratory Animal Science of Suranaree University of Technology, Thailand. Healthy male (260–290 g) and female (190–220 g) Wistar rats were bred in the Experimental Animal laboratory, Suranaree University of Technology, Nakhon Ratchasima Province, Thailand. They were acclimatized to the laboratory environment for one week prior to experimentation. The rats were randomly assigned to three groups (five males, five females per group). The first group fed a basal diet was defined as the control group. The second and third groups were fed daily with diets containing CDF at 2.5% and 15% (*w*/*w*), respectively. Animals were housed individually in screen-bottomed, stainless steel cages in a room maintained at 22 ± 3 °C with a 12 h light/dark cycle. In the experimental period, food and water were provided ad libitum. Food consumption was recorded daily, but body weights were recorded every two days. The rats were examined daily for clinical signs of toxicity, such as mortality, respiratory pattern, changes in general behavior, skin, eyes, fur and somatomotor activity. After the experimental period (14 days), the rats were sacrificed with carbon dioxide after fasting for 12 h. Blood samples were collected via cardiac puncture, and serum was prepared for biochemical tests. The effects of DF in all groups on body weight, relative organ weight, food consumption, and organ histopathology were examined. All procedures were performed in accordance with the Guidelines of the Animal Care and Use of Laboratory Animals from the Center for Laboratory Animal Science of Suranaree University of Technology.

#### 2.3.3. Hematological Assay

The rats were fasted overnight before blood collection. Blood samples were drawn and transferred into separate tubes containing ethylenediaminetetraacetic acid (EDTA). An XS-800i Sysmex (Sysmex Corporation, Kobe, Japan) was used to measure hematocrit (HCT), red blood cells (RBCs), hemoglobin (HGB), mean corpuscular volume (MCV), mean corpuscular hemoglobin (MCH), mean corpuscular hemoglobin concentration (MCHC), total leukocyte count (WBC), lymphocytes, monocytes, eosinophils, basophils, platelet count (PLT), and polymorph nuclear neutrophils (PMNs).

#### 2.3.4. Blood Chemistry

Blood samples were centrifuged to separate plasma. Clinical chemistry parameters were analyzed using the VITROS^®^ 5600 Chemistry System (Ortho-Clinical Diagnostics, Inc., New York, NY, USA) for quantification of alanine aminotransferase (ALT), aspartate aminotransferase (AST), alkaline phosphatase (ALP), glucose (GLU), total cholesterol (TC), triglycerides (TG), creatinine (CREA), and blood urea nitrogen (BUN).

#### 2.3.5. Organ Weights

The liver, heart, lungs, kidneys, adrenal glands, spleen, and testes/ovaries organs were clipped of any adherent tissue, as appropriate, and washed with normal saline solution to eliminate blood from the organs. Paired organs were weighed individually. Relative organ weights (%) were calculated against fasting body weight.

#### 2.3.6. Histopathological Examination

The liver was dissected, weighed, and then immersed immediately in neutral buffer formalin (formaldehyde neutral buffer: 10% formalin 238 mL, distilled water 762 mL, pH 7.4) for no more than 24 h. Liver sections were dehydrated and then embedded in paraffin. Sections with a thickness of 5 mm were cut, deparaffinized, rehydrated, and stained with hematoxylin–eosin (H&E). The sections were subsequently subjected to photomicroscope examination (Nikon Corporation, Tokyo, Japan) coupled with Olympus DP72 (Hollywood International group, Bangkok, Thailand).

### 2.4. Cholesterol-Lowering Properties of Dietary Fiber

#### 2.4.1. Diet Preparation

The experiments were divided into five groups: Group 1 was fed a basal diet (Normal), which was prepared by C.P. Perfect Companion Group, Thailand (No. 082G/15); Group 2 received a high-fat diet without fiber (Control); Group 3 received a high-fat diet without fiber together with gavage of 10 mg/kg/day simvastatin (Simvastatin); Group 4 received a high-fat diet containing 5% (*w*/*w*) cassava dietary fiber (CDF); and Group 5 received a high-fat diet containing 5% (*w*/*w*) cellulose (Cellulose). The diets of Groups 2 to 5 were prepared by Research Diets, Inc. (New Brunswick, NJ, USA) and supplemented with cholesterol (1.25 g/100 g) and sodium cholic acid (0.5 g/100 g) to induce hypercholesterolemia in Wistar rats. The ingredients and chemical composition of the diets are listed in Table 1.

#### 2.4.2. Experimental Design

Thirty male rats (250–290 g) were obtained from the Laboratory Animal Center of Suranaree University of Technology, Thailand. After an acclimatization period of 7 days, the rats were randomly allotted to the five diet groups with six animals in each group. Animals were housed in individual stainless-steel cages kept in a room maintained at 22 ± 3 °C with a 12 h light/dark cycle. For the whole experimental period (30 days), food and water were provided ad libitum. In addition, feces were collected and weighed daily, and food intakes and body weights were recorded every day. Fecal samples were lyophilized, weighed, milled, and stored at −20 °C until analysis. Before the experiment, blood samples were taken from the rats’ tail veins. At the end of the experiment, the rats were sacrificed using carbon dioxide after fasting for 12 h. Blood was drawn from the left atrium of the heart, and serum was prepared for biochemical tests. The livers were removed, weighed, and kept at −80 °C before pathological analysis. All procedures were performed in accordance with the Guidelines of the Animal Care and Use of Laboratory Animals from the Center for Laboratory Animal Science of Suranaree University of Technology, Thailand.

#### 2.4.3. Biochemical Assays of Serum Cholesterol, Triglyceride, and Glucose

The concentrations of total cholesterol, high-density lipoprotein (HDL) cholesterol, low-density lipoprotein (LDL) cholesterol, triglyceride, and glucose in the serum samples were quantified enzymatically via the VITROS^®^ 350 Chemistry System (Ortho-Clinical Diagnostics, Inc., Raritan, NJ, USA) at the Biochemical Laboratory of Suranaree University of Technology Hospital.

#### 2.4.4. Liver Total Lipids and Cholesterol

Following the method described by the authors of [13], total lipids were extracted from livers (1–2 g) with a chloroform/methanol mixture (2:1 *v*/*v*). The concentration of total lipids in the liver tissue was determined gravimetrically by removing the organic solvents in the liver lipid extract. The liver cholesterol concentration in the liver lipid extract was determined through colorimetric analysis at a wavelength of 490 nm [14].

#### 2.4.5. Fecal Total Lipids and Cholesterol

The total fecal lipids and cholesterol in dried fecal samples were extracted with a chloroform/methanol mixture (2:1 *v*/*v*) according to the method of [13]. The total lipid in the dried fecal sample was then quantified gravimetrically by evaporating the organic solvent in the fecal lipid extract. The concentration of cholesterol in the fecal lipid extract was determined colorimetrically at 490 nm [14].

#### 2.4.6. Determination of Short-Chain Fatty Acids (SCFAs) in Feces

Short-chain fatty acids in feces were analyzed with slight modifications following the method of [15]. The dried feces (150 mg) were homogenized in 2.5 mL of methanol, and the mixture was incubated for 15 min. After filtering the samples through a 0.45 m membrane, they were transferred to a vial. Following this step, aliquots (1 µL) were injected into a gas chromatograph (Hewlett Packard HP 6890 series GC system, Santa Clara, CA, USA), equipped with an Agilent J&W GC capillary column (DB-FFAP 30 m; ID: 0.32 mm; film: 0.25 µm; Agilent Technologies, Santa Clara, CA, USA). The chromatographic conditions were as follows: injector and detector temperature of 250 °C and a flow of 1.8 mL/min; the oven temperature was set to 80 °C during the first 5 min and was increased to 170 °C at a rate of 10 °C/min; the temperature was maintained at 170 °C for 1 min and was increased to 250 °C at a rate of 30 °C/min; and the temperature was then maintained at 250 °C for 5 min. Helium was used as a carrier gas, and the split ratio was 30:1. The SCFAs were quantitatively determined via a comparison of the retention times and peak areas of standards (acetic acid, propionic acid, and butyric acid). All samples were analyzed in triplicate.

### 2.5. Statistical Analysis

All experiments were performed in triplicate, and mean values (on a dry basis) with standard deviations are reported. The experimental data were analyzed using an analysis of variance (ANOVA). SPSS^®^ software version 17 (SPSS Inc., Chicago, IL, USA) was used to perform all statistical calculations.

## 3. Results

### 3.1. Chemical Compositions

The proximate analysis of cassava pulp powder and CDF is shown in Table 2 and Table 3. The crude fiber, carbohydrate, starch, NDF, ADF, ADL, cellulose, and hemicellulose contents of cassava pulp were 17.23%, 70.15%, 58.11%, 31.40%, 25.08%, 4.16%, 20.92%, and 6.32%, respectively. Additionally, the composition of the dietary fiber containing crude fiber, carbohydrate, starch, NDF, ADF, ADL, cellulose, and hemicellulose was 40.24%, 48.46%, 8.50%, 79.03%, 70.14%, 58.55, and 8.89%, respectively. The main component of cassava pulp and CDF was high crude fiber.

### 3.2. Short-Term Toxicity of Dietary Fiber

#### 3.2.1. Clinical Observation, Feed Consumption, and Body Weight

In this trial, a comparison was made between a control group and diets containing 2.5% and 15% (*w*/*w*) CDF. After 14 days of oral treatment with 2.5 and 15% (*w*/*w*) CDF, rats moved freely and behaved properly, including normal feces hardness and color. Furthermore, there was no diarrhea, constipation, stomach bulge, or sunken stomach, and the rats’ fur were smooth, with no signs of skin laxation, wrinkles, redness, or other occurrences. Furthermore, no mortality of any rats and no symptoms of poisoning were observed from the morphological observation throughout the experiments (Table 4). There was no abnormal behavior or mortality of rats in this trial, implying that CDF was safe for rats.

Table 5 shows the average daily consumption and body weight gain of the rats. The control group consumed the most food and was substantially different (*p* < 0.05) between meals containing 2.5% and 15.0% (*w*/*w*) of CDF in both sexes. It is possible that the high DF level causes hardness and a change in food quality. The increase in male rat body weight was similar in all groups after 7 and 14 days. While female rats’ body weight gain was not different in all groups after 7 days, it was lower in the 15% group after 14 days (*p* < 0.05).

#### 3.2.2. Hematology Parameters

Almost all hematological parameters (Table 6) of both male and female rats in each dosing group did not indicate significant differences (*p* < 0.05) when compared to the control group throughout the experiments. These findings demonstrated that CDF has no effect on rat hematological parameters.

#### 3.2.3. Blood Chemistry

The serum biochemical characteristics of male and female rats were also examined (Table 7). Serum total cholesterol in male rats was considerably lower in the 2.5% group than in the control group (*p* < 0.05). Triglyceride levels in the 15.0% group were also considerably lower than those in the control group (*p* < 0.05). Female rats in the 2.5% group had lower BUN and serum triglyceride levels than the control group (*p* < 0.05). Triglycerides in the 15.0% group were significantly lower than those in the control group (*p* < 0.01). The data above showed that CDF had no harmful effect on rats. Furthermore, CDF has been shown to lower triglyceride, cholesterol, and glucose levels in the blood. It is possible that dietary fiber obtained from cassava pulp could reduce the risk of cardiovascular disease and diabetes in people.

#### 3.2.4. Organ Weights

Table 8 shows the relative organ weights of male and female rats. The results revealed that the relative organ weight of male rats in both groups was not substantially different for the lung, heart, liver, spleen, left adrenal gland, and testis (*p* > 0.05), but the right kidney was significantly different between CDF diets and the control group (*p* < 0.05). The relative organ weights of female rats in both groups were not substantially different for the lung, heart, liver, spleen, adrenal glands, and ovaries (*p* > 0.05). The findings indicated that CDF had no harmful effect on all visceral organs of rats.

#### 3.2.5. Histopathological Examination

**Figure 1:**
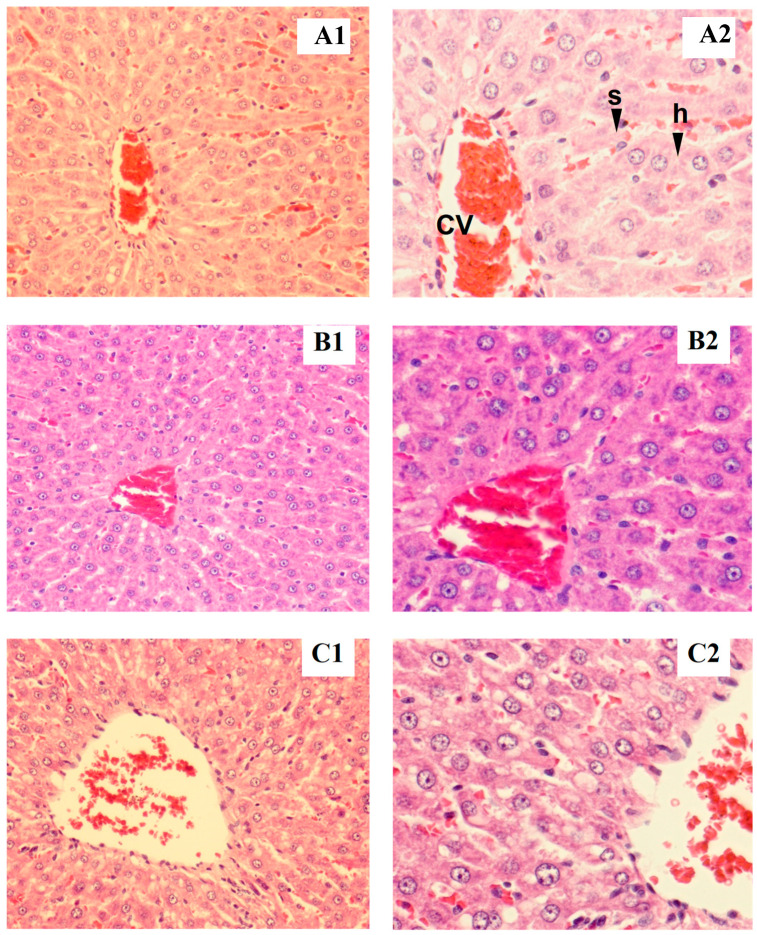
Tissue section of male liver rat: control (**A1**,**B1**), low dose (**A2**,**B2**), high dose (**C1**,**C2**), h = hepatocyte, cv = central vein, s = sinusoid (magnified ×200 for **A1**–**C1** and ×400 for **A2**–**C2**).

**Figure 2:**
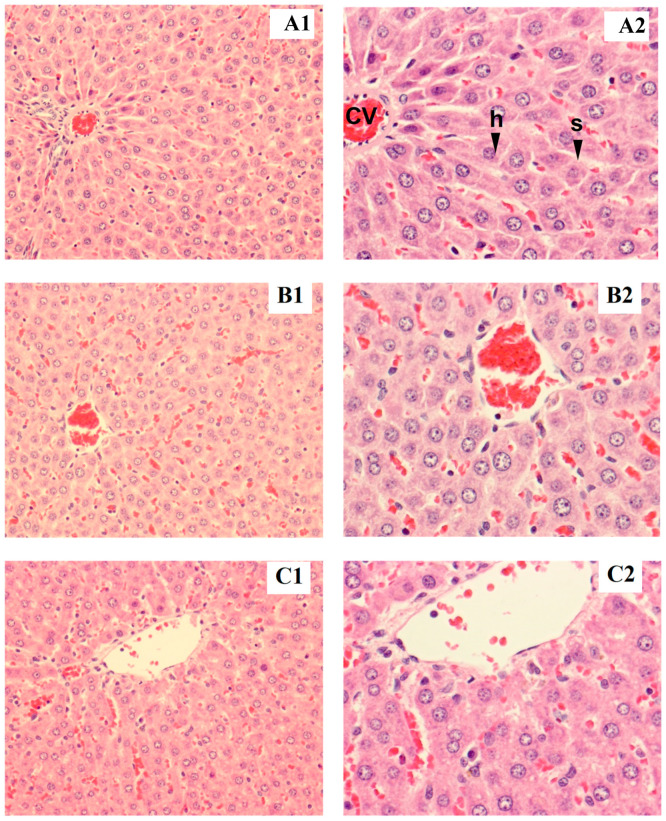
Tissue section of female liver rat: control (**A1**,**B1**), low dose (**A2**,**B2**), high dose (**C1**,**C2**), h = hepatocyte, cv = central vein, s = sinusoid (magnified ×200 for **A1**–**C1** and ×400 for **A2**–**C2**).

Rat liver samples were cut into slices and stained for histological evaluation (Figure 1 and Figure 2). The internal structure of hepatocytes was well-preserved in both male and female rats, with no evidence of cell enlargement, inflammatory cell infiltration, degeneration, or necrosis. The hepatic lobular structure was normal and transparent, with no enlargement of the central vein or sinusoids.

### 3.3. Cholesterol-Lowering Properties of Dietary Fiber

#### 3.3.1. Food Intake, Body Weight Gain, and Fresh Fecal Mass

This investigation compared the normal group (rats fed a basal diet) to the group of rats fed high-fat diets to develop hypercholesterolemia. This diet included a fiber-free diet (control), fiber-free meals combined with simvastatin (10 mg/kg/day), a commercial drug used to lower cholesterol levels, a 5% cassava dietary fiber (CDF)-containing diet, and a 5% cellulose-containing diet. Table 9 shows the rats’ food consumption, body weight increase, and fresh fecal mass. The rats behaved vigorously and healthily throughout.

The experiment, according to daily observations. The normal group consumed the most after 30 days of feeding; however, there was no difference between the CDF and cellulose groups (*p* > 0.05). The control and simvastatin groups, on the other hand, consumed significantly less food than the other groups (*p* < 0.05). The results reveal that the experimental diets with and without fiber have equal food intake results. When compared to the other groups, the simvastatin group acquired considerably less body weight (*p* < 0.05). This could be due to the negative effects of simvastatin and daily gavage, which could cause stomach irritation in the rats’ eating behavior. The fecal bulk of rats fed a high-fat diet was considerably smaller than that of the control group (*p* < 0.05). The findings imply that a high-fat diet may affect the excretory system of rats.

#### 3.3.2. Biochemical Assays of Serum Cholesterol, Triglyceride, and Glucose

Rat serum total cholesterol, triglyceride, HDL cholesterol, LDL cholesterol, and glucose concentrations were measured, as indicated in Table 10. The initial serum total cholesterol level was 68.33–84 mg/dL, the serum triglyceride level was 69.50–89.83 mg/dL, the HDL cholesterol level was 46.17–55.0 mg/dL, and the glucose level was 98.50–115.17 mg/dL. At the end of the feeding period (30 days), there was a significant (*p* < 0.05) difference in serum total cholesterol and HDL cholesterol levels between the normal and high-fat diet groups. When compared to the control group, the consumption of CDF and simvastatin significantly (*p* < 0.05) reduced serum total cholesterol levels by 9.18% and 11.0%, respectively (Table 5), but the consumption of cellulose revealed no significant difference in total cholesterol levels. These findings show that incorporating CDFs into meals can successfully lower serum total cholesterol levels (with the hypocholesterolemic effect of CDFs being significantly stronger than that of cellulose but most similar to that of simvastatin). Other agricultural products containing dietary fiber (such as sugar beet pulp, apple pomace, sweet orange peel, and carrot pomace, among others) may also have hypocholesterolemic and hypolipidemic qualities. When compared to the control group, the simvastatin, CDF, and cellulose groups showed a significant decrease (*p* < 0.05) in the level of serum triglycerides by 49.0%, 51.3%, and 49.0%, respectively. The CDF group maintains HDL cholesterol levels at the same level as the simvastatin and cellulose groups (*p* < 0.05) while having a higher HDL cholesterol content than the control group (*p* < 0.05). In this study, the results show a 51.3% reduction in the serum triglyceride level (Table 10), which is possibly due to the reduced adsorption of VLDL and LDL, leading to lower levels of circulating forms of triglycerides. As shown in Table 5, there was no significant (*p* > 0.05) difference in the glucose contents of the CDF, normal, control, and cellulose groups. In contrast, the simvastatin group had a lower glucose level than the other groups (*p* < 0.05).

#### 3.3.3. Total Lipid Content in Liver and Feces

**Figure 3:**
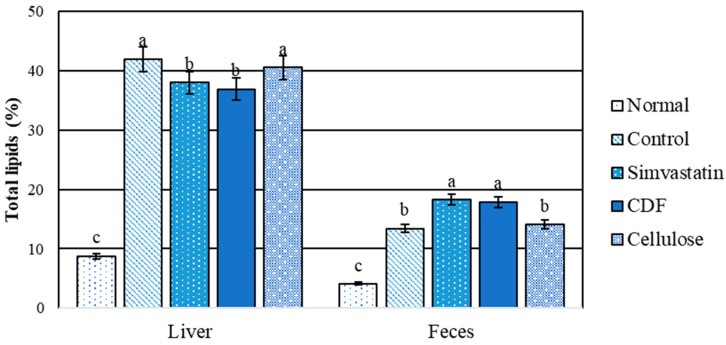
Total lipids content (%) in liver and feces of male rats fed various diets for 30 days. Group means with different superscripted letters in the same set show significant differences (*p* < 0.05).

A comparison between the normal and hyperlipidemic groups for liver and fecal total lipids is shown in Figure 3. The results indicate that there is a significant difference (*p* < 0.05) between the control and CDF groups in the total amount of lipids (both groups had shown a high content of total lipids in rats that were fed high fat diets for 30 days). As a result, high-fat diets cause fat storage in rat livers. Total lipid levels in the CDF and simvastatin groups were significantly (*p* < 0.05) lower than total lipid levels in the control and cellulose groups. As a result, CDF reduces the buildup of lipids in the livers of rats fed a high-fat diet, demonstrating a hepatoprotective effect. In terms of feces, no significant changes were seen between the CDF and simvastatin groups, which had greater total lipid levels than the other groups. The findings suggest that CDF increases fat excretion via feces.

#### 3.3.4. Cholesterol Content in Liver and Feces

**Figure 4:**
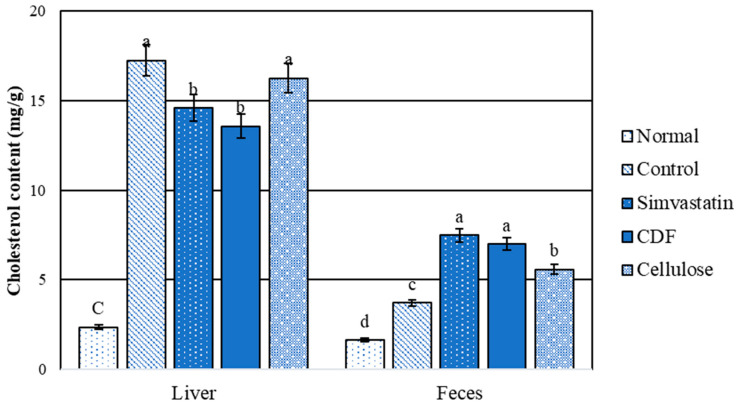
Cholesterol content (mg/g) in liver and feces of male rats fed various diets for 30 days. Group means with different superscripted letters in the same set show significant differences (*p* < 0.05).

The comparison between the normal and hyperlipidemic groups for liver and fecal cholesterols is shown in Figure 4. The results indicate that the normal group had significantly (*p* < 0.05) lower cholesterol contents than the high-fat diet rat group. The level of liver cholesterol obtained from the CDF and simvastatin groups was significantly (*p* < 0.05) lower than that for the control and cellulose groups. There was no significant difference in stool cholesterol content between the CDF and simvastatin groups. Furthermore, the control and cellulose groups had lower fecal cholesterol levels than the CDF and simvastatin groups. When compared to the control, the results show that CDF lowers serum triglycerides and total cholesterol (Table 10), which is consistent with total liver lipids and cholesterol content (Figure 3 and Figure 4). As the data show a higher cholesterol content in the feces, this could be attributable to an increased excretion of cholesterol and lipids in stools.

#### 3.3.5. Short-Chain Fatty Acids in Feces

Colonic microflora normally use carbohydrates as a substrate for fermentation, and the main fermentation products are short-chain fatty acids (SCFAs: acetic acid, propionic acid, and butyric acid) and gases (CO_2_, CH_4_, and H_2_). These end-products are excreted in the stool or absorbed from the colon [16]. As expected, acetic acid was the predominant fatty acid in the feces of all the animals [17,18]. Acetic acid was also the most frequently found chemical in this study (Table 6). The CDF group had the highest contents of acetic acid, propionic acid, and butyric acid. However, there were no significant differences from the normal, control, and simvastatin groups. The cellulose group, on the other hand, had the lowest level of short-chain fatty acid content (significance, *p* < 0.05). This could be because cellulose is an insoluble dietary fiber with low fermentability and prebiotic characteristics. The results of short-chain fatty acids of the CDF group are shown in Table 11.

## 4. Discussion

The cassava pulp in this study still contained high starch contents, while the dietary fiber contained high cellulose in an insoluble form. It was clear that the main constituents of cassava pulp and dietary fiber are carbohydrates. According to numerous studies, cassava pulp is mostly composed of starch, followed by cellulose, hemicellulose, and lignin [19,20,21,22]. The cellulose content of cassava pulp fiber was higher than that of other food industry wastes, such as malt bagasse, oat hull, rice hull, and fibrous residue of banana pseudosystems, but it was lower in hemicellulose and lignin content [23]. Furthermore, due to the hydrolysis properties of the enzymes utilized, dietary fiber revealed greater levels of NDF, ADF, ADL, cellulose, and hemicellulose when it was extracted. For purification from starch, thermostable amylase was heated in cassava pulp solution to release bound starch granules. This enzyme hydrolyzes the α-1,4 glycosidic bond of starch via random cleavage but does not hydrolyze the α-1,6 glycosidic bond of amylopectin in starch granules. Amyloglucosidase was used to hydrolyze α-1,4 glycosidic bonds from the nonreducing end of starch. This enzyme could hydrolyze the α-1,6 glycosidic bond of amylopectin at a slow rate; however, the protein in the raw material was eliminated by neutrase [24]. Lacourse et al. [25] reported that reducing starch content from tapioca fiber via enzymatic treatment, particularly when performed with alpha-amylase, can reduce starch content from approximately 60% to 15%, preferably 5%, by weight. According to the NDF criteria, cassava pulp fiber contained a high concentration of insoluble dietary fiber, specifically cellulose, hemicellulose, and lignin. 

After the oral administration of CDF meals, no mortality or poison symptoms were observed via morphological observation throughout this study in short-term toxicity research. There was no abnormal behavior or mortality of rats in this trial, implying that DF derived from cassava pulp was safe for rats. Hong et al. [26] investigated the acute and long-term toxicity of DF derived from wheat bran, which was employed as a food ingredient in cookies. The results revealed that there were no deaths in any dose group, which indicates that the oral LD50 of wheat bran DF for male and female mice is more than 21.5 g/kg. They suggested that DF cookies were nontoxic. Rats’ daily consumption and body weight increase were compared in both sexes between diets containing 2.5% and 15.0% (*w*/*w*) CDF. The increase in male rat body weight was similar in all groups after 7 and 14 days. These findings validated recent findings by the authors of [27], who discovered that female rats fed diets containing 5% (*w*/*w*) rice hull fiber had lower body weights than the other groups (0%, 2.5%, and 3.75%). They proposed that dietary fiber could aid in weight loss. Because it does not contribute energy, it reduces the energy density of foods; as a result, body weight is reduced. Overall, the body weight of male and female rats at 14 days increased normally when compared to the control group, demonstrating that CDF may not have any specific impacts on rat growth. A prior study by the authors of [28] demonstrated that the reduction in total cholesterol may be related to the LDL and VLDL cholesterol fractions and that the CDF group may have a fermentative potential. 

Finally, the consumption of fibers could promote hypolipidemic and hypocholesterolemic actions through a combination of physiological effects, including decreased transit time, a reduced rate of lipids and cholesterol absorption, higher bile acid adsorption, increased cholesterol catabolism to bile acids, and retarded cholesterol biosynthesis [28,29,30].

The study on the safety and cholesterol-lowering effects of cassava pulp dietary fiber (CDF) in Wistar rats is a promising start, but further research is needed to confirm the safety and efficacy of CDF in humans and to develop food products that incorporate CDF.

Specifically, researchers should conduct clinical trials on humans to confirm the safety and efficacy of CDF in lowering cholesterol, investigate the long-term effects of CDF consumption, and develop food products that incorporate CDF and that are palatable and convenient for consumers to consume. In addition, researchers should investigate the mechanism by which CDF lowers cholesterol, the optimal dosage of CDF for cholesterol-lowering effects, and the effects of CDF on other blood lipid levels, such as HDL cholesterol and LDL cholesterol, and other aspects of cardiovascular health, such as blood pressure and inflammation. By investigating these additional factors, researchers can gain a better understanding of the potential of CDF as a cholesterol-lowering agent and develop strategies for its safe and effective use in humans.

## 5. Conclusions

In conclusion, this study has demonstrated the safety and cholesterol-lowering effects of cassava pulp dietary fiber (CDF) in Wistar rats. CDF was found to be non-toxic at a dose of 15% in both male and female rats. Moreover, CDF was shown to be effective in lowering serum triglyceride, serum total cholesterol, total liver lipids, and liver cholesterol, while also increasing the excretion of lipids and cholesterol via stools. These effects were comparable to those of simvastatin, a commonly prescribed cholesterol-lowering drug.

Based on these findings, CDF has the potential to be used as a functional food ingredient for the prevention and management of high cholesterol. However, further research is needed to investigate the long-term effects of CDF consumption and to develop food products that incorporate CDF.

Overall, this study provides valuable insights into the potential of CDF as a safe and effective cholesterol-lowering agent.

## Figures and Tables

**Table 1:** Formulations of the experimental diets.

Ingredients ^a^	Control (Fiber Free)	Cassava DF	Cellulose
Casein	140	140	140
DL-methionine	2	2	2
Corn starch	435	435	435
Maltodextrin	150	150	150
Sucrose	100	100	100
Cassava DF	-	50	-
Cellulose	-	-	50
Soybean oil	50	50	50
Coconut oil	35	35	35
Mineral mix S10001	35	35	35
Calcium carbonate	5.5	5.5	5.5
Sodium chloride	8	8	8
Potassium citrate	10	10	10
Vitamin mix V10001	10	10	10
Choline bitartrate	2	2	2
Cholesterol	12.5	12.5	12.5
Sodium cholic acid	5	5	5
FD&C blue dye #1	0.1	-	-
FD&C yellow dye #5	-	0.1	-
FD&C red dye #40	-	-	0.1

^a^ The ingredients are expressed as g/kg of diet (dry weight).

**Table 2:** Chemical composition (%) of cassava pulp powder and dietary fiber (dry weight basis).

Components	Cassava Pulp (%)	Dietary Fiber (%)
Crude protein	2.02 ± 0.19	1.01 ± 0.10
Fat	0.21 ± 0.08	0.25 ± 0.06
Moisture	6.63 ± 0.11	5.52 ± 0.09
Ash	3.76 ± 0.05	4.52 ± 0.04
Crude fiber	17.23 ± 0.13	40.24 ± 2.22
Carbohydrate	70.15	48.46
Starch	58.11 ± 0.06	8.50 ± 0.31
Neutral detergent fiber (NDF)	31.40 ± 0.58	79.03 ± 0.51
Acid detergent fiber (ADF)	25.08 ± 0.17	70.14 ± 0.40
Acid detergent lignin (ADL)	4.16 ± 0.10	11.59 ± 0.01
Cellulose ^a^	20.92	58.55
Hemicellulose ^b^	6.32	8.89

^a^ ADF-ADL, ^b^ NDF-ADF.

**Table 3:** Functional properties of dietary fiber and cellulose.

Functional Properties	Dietary Fiber	Cellulose
Water-holding capacity (g/g dry weight)	8.17 ± 0.40	4.92 ± 0.33
Water retention capacity (g/g dry weight)	8.36 ± 0.20	5.95 ± 0.49
Swelling capacity (mL/g)	4.82 ± 0.15	1.13 ± 0.15
Oil-holding capacity (g/g)	3.97 ± 0.14	2.66 ± 0.09

**Table 4:** Death rate of rats in control group and rats treated with diets containing 2.5% and 15.0% (*w*/*w*) of CDF for 14 days.

Group/Dose % (*w*/*w*)	Death Rate ^a^		Side Effect
	Male	Female	
Control	0/5	0/5	No ^b^
2.5	0/5	0/5	No
15.0	0/5	0/5	No

^a^ Death rate = Amount of rat dies in this experiment/total rats in this experiment. ^b^ No = No side effect during experiment.

**Table 5:** Average daily consumption and body weight gain of rats treated with diets containing 2.5% and 15.0% (*w*/*w*) of CDF for 14 days.

Sex	Group/Dose % (*w*/*w*)	Consumption (g/day)	Body Weight Gain (g)	
			Day 7	Day 14
Male	Control	21.04 ± 1.46	21.33 ± 1.15	20.33 ± 1.53
	2.5	20.32 ± 1.50 *	21.20 ± 1.79	18.20 ± 2.17
	15.0	19.95 ± 1.65 *	18.60 ± 4.83	15.40 ± 4.04
Female	Control	17.85 ± 1.21	12.80 ± 2.28	12.40 ± 2.51
	2.5	17.16 ± 1.43 *	13.80 ± 2.17	11.00 ± 2.24
	15.0	17.11 ± 1.34 *	12.00 ± 2.74	8.60 ± 1.67 *

Values were means ± SD. * *p* < 0.05 vs. control group.

**Table 6:** Hematological parameters in rats treated with diets containing 2.5% and 15.0% (*w*/*w*) of CDF for 14 days.

Parameters	Relative Organ Weight (g/100 g)					
	Group/Dose (% *w*/*w*)					
	Male			Female		
	Control	2.5	15.0	Control	2.5	15.0
Hb (g/dL)	16.60 ± 1.60	16.38 ± 0.79	16.34 ± 0.79	16.80 ± 1.32	16.03 ± 1.32	17.12 ± 0.86
Hct (%)	49.87 ± 4.06	49.30 ± 2.03	49.18 ± 2.78	48.90 ± 3.65	47.28 ± 2.78	50.10 ± 2.62
WBC (×10^3^ cell/mm^3^)	6.91 ± 2.13	6.26 ± 1.88	6.31 ± 3.53	5.84 ± 1.97	6.15 ± 1.61	8.72 ± 3.41
PMNs (%)	22.67 ± 8.08	18.00 ± 8.49	17.60 ± 4.56	22.80 ± 4.60	23.50 ± 9.29	16.80 ± 5.59
Lymphocyte (%)	72.67 ± 7.02	74.80 ± 6.42	77.40 ± 2.41	72.40 ± 5.18	72.00 ± 9.93	82.40 ± 5.18
Monocyte (%)	4.00 ± 0	5.00 ± 1.15	3.80 ± 2.28	4.00 ± 1.41	4.50 ± 1.91	3.60 ± 1.67
Eosionphil (%)	1.33 ± 2.31	2.40 ± 0.89	0.80 ± 1.10	1.60 ± 1.67	1.50 ± 1.00	1.20 ± 1.10
MCV (fl.)	54.73 ± 2.40	55.26 ± 2.60	54.36 ± 2.39	53.84 ± 0.74	53.78 ± 1.52	53.10 ± 1.15
MCH (pg.)	18.23 ± 0.55	18.34 ± 0.75	18.04 ± 0.46	18.48 ± 0.28	18.20 ± 0.34	18.14 ± 0.36
MCHC (%)	33.23 ± 0.51	33.22 ± 0.37	33.26 ± 0.76	34.36 ± 0.56	33.88 ± 0.43	34.16 ± 0.22
Platelet count (×10^3^ cell/mm^3^)	832.67 ± 84.01	847.20 ± 44.55	778.20 ± 113.2	804.04 ± 117.1	742.50 ± 154.31	962.40 ± 7.27
RBC count	9.14 ± 1.15	8.94 ± 0.65	9.06 ± 0.60	8.09 ± 2.71	8.81 ± 0.75	9.43 ± 0.35

Values were means ± SD, *p* < 0.05 vs. control group.

**Table 7:** Serum biochemical parameters in rats treated with diets containing 2.5% and 15.0% (*w*/*w*) of CDF for 14 days.

Parameters	Relative Organ Weight (g/100 g)					
	Group/Dose (% *w*/*w*)					
	Male			Female		
	Control	2.5	15.0	Control	2.5	15.0
ALP (U/L)	95.00 ± 17.69	120.00 ± 19.76	114.60 ± 16.09	65.60 ± 10.33	74.00 ± 20.74	60.40 ± 11.01
ALT (U/L)	43.67 ± 24.99	24.80 ± 4.55	28.20 ± 2.28	24.60 ± 3.97	26.40 ± 7.44	19.80 ± 3.83
AST (U/L)	126.00 ± 15.00	108.80 ± 17.57	120.80 ± 47.92	146.00 ± 44.82	228.00 ± 96.29	179.60 ± 70.22
BUN (mg/dL)	20.33 ± 1.15	20.00 ± 3.54	18.80 ± 1.30	27.40 ± 3.78	21.20 ± 2.39 *	23.80 ± 5.07
Creatinine (mg/dL)	0.54 ± 0.02	0.50 ± 0.04	0.51 ± 0.06	0.72 ± 0.10	0.62 ± 0.08	0.71 ± 0.07
TC (mg/dL)	82.33 ± 3.51	69.60 ± 4.83 *	75.40 ± 7.99	72.80 ± 9.83	63.20 ± 10.08	65.60 ± 10.43
TG (mg/dL)	108.00 ± 34.22	83.80 ± 12.77	71.00 ± 13.00 *	116.60 ± 16.24	82.40 ± 26.71 *	64.00 ± 7.31 **
Glucose (mg/dL)	326.00 ± 56.67	275.20 ± 93.18	305.20 ± 58.0	185.20 ± 85.51	160.40 ± 68.91	131.80 ± 38.66

Values were means ± SD. * *p* < 0.05 vs. control group. ** *p* < 0.01 vs. control group.

**Table 8:** Visceral organ weights of male and female rats treated with diets containing 2.5% and 15.0% (*w*/*w*) of CDF for 14 days.

Organs		Relative Organ Weight (g/100 g)					
		Group/Dose (% *w*/*w*)					
		Male			Female		
		Control	2.5	15.0	Control	2.5	15.0
Lung		0.43 ± 0.03	0.51 ± 0.06	0.46 ± 0.08	0.59 ± 0.07	0.54 ± 0.04	0.62 ± 0.04
Heart		0.33 ± 0.02	0.33 ± 0.01	0.34 ± 0.05	0.40 ± 0.04	0.37 ± 0.04	0.38 ± 0.02
Liver		2.96 ± 0.15	3.04 ± 0.04	3.04 ± 0.09	2.98 ± 0.25	3.26 ± 0.30	3.09 ± 0.12
Spleen		0.22 ± 0.03	0.22 ± 0.03	0.23 ± 0.06	0.29 ± 0.02	0.30 ± 0.02	0.29 ± 0.03
Kidney	L	0.32 ± 0.02	0.33 ± 0.02	0.33 ± 0.02	0.35 ± 0.02	0.35 ± 0.01	0.34 ± 0.03
	R	0.30 ± 0.00	0.33 ± 0.03 *	0.33 ± 0.02 *	0.34 ± 0.01	0.33 ± 0.02	0.34 ± 0.03
Adrenal gland	L	0.02 ± 0.00	0.01 ± 0.00	0.01 ± 0.00	0.03 ± 0.01	0.03 ± 0.01	0.03 ± 0.01
	R	0.01 ± 0.00	0.02 ± 0.00	0.01 ± 0.00	0.03 ± 0.00	0.03 ± 0.00	0.03 ± 0.01
Testis ^a^/Ovaries ^b^	L	0.51 ± 0.09	0.61 ± 0.06	0.57 ± 0.04	0.03 ± 0.01	0.03 ± 0.01	0.03 ± 0.01
	R	0.50 ± 0.11	0.60 ± 0.07	0.58 ± 0.03	0.03 ± 0.00	0.03 ± 0.01	0.03 ± 0.01

Values were means ± SD. ^a^ Testis for male rats; ^b^ Ovaries for female rats. * *p* < 0.05 vs. control group.

**Table 9:** Food intake (average daily consumption), body weight gain, and fresh fecal mass (g) of male rats fed various diets for 30 days.

Parameter	Group (Mean ± S.D.)				
	Normal	Hyperlipidemia Rats			
		Control	Simvastatin	CDF	Cellulose
Food intake (g/day)	20.26 ± 2.71 ^a^	18.45 ± 4.00 ^b^	16.48 ± 3.13 ^c^	19.60 ± 3.90 ^a^	19.72 ± 3.22 ^a^
Body weight gain (g)	97.50 ± 7.58 ^a^	99.17 ± 18.0 ^a^	65.83 ± 13.57 ^b^	96.67 ± 13.66 ^a^	100 ± 7.89 ^a^
Fecal mass (g/day)	4.68 ± 1.38 ^a^	2.35 ± 2.12 ^b^	2.47 ± 2.53 ^b^	2.76 ± 1.27 ^b^	3.07 ± 1.28 ^b^

Means with different superscripted letters in the same row show significant differences (*p* < 0.05).

**Table 10:** The concentration of serum total cholesterol, triglyceride, HDL cholesterol, LDL cholesterol, and glucose of male rats on day 1 and day 31.

Parameter (mg/dL)	Group (Mean ± S.D.)				
	Normal	Hyperlipidemic Rats			
		Control	Simvastatin	CDF	Cellulose
At day 0					
Total cholesterol	84.0 ± 3.46 ^a^	80.67 ± 6.86 ^a^	82.17 ± 12.25 ^a^	73.83 ± 9.15 ^ab^	68.33 ± 8.7 ^b^
Triglyceride	82.50 ± 12.50 ^ab^	69.50 ± 5.09 ^c^	84.17 ± 13.21 ^a^	89.83 ± 5.15 ^a^	88.33 ± 7.06 ^a^
HDL cholesterol	55.0 ± 6.0 ^a^	52.67 ± 3.14 ^ab^	54.83 ± 7.55 ^a^	49.33 ± 6.31 ^ab^	46.17 ± 5.71 ^b^
LDL cholesterol	<30	<30	<30	<30	<30
Glucose	115.17 ± 4.75 ^a^	99.83 ± 5.98 ^bc^	98.50 ± 12.66 ^c^	110.33 ± 10.01 ^ab^	100.67 ± 8.12 ^bc^
At day 31st					
Total cholesterol	77.67 ± 6.59 ^c^	147.0 ± 12.44 ^a^	130.83 ± 15.28 ^b^	133.50 ± 4.93 ^b^	141.17 ± 9.20 ^ab^
Triglyceride	97.0 ± 12.38 ^b^	187.17 ± 11.32 ^a^	95.67 ± 9.33 ^b^	91.17 ± 8.28 ^b^	95.50 ± 7.20 ^b^
HDL cholesterol	43.33 ± 7.66 ^a^	28.17 ± 6.91 ^c^	36.67 ± 3.67 ^b^	35.17 ± 4.22 ^b^	32.83 ± 4.79 ^bc^
LDL cholesterol	<30	<30	<30	<30	<30
Glucose	189.50 ± 19.59 ^b^	212.17 ± 13.36 ^a^	171.33 ± 8.19 ^c^	203.83 ± 4.96 ^ab^	202.33 ± 10.33 ^ab^

Means with different superscripted letters in the same row differ significantly (*p* < 0.05).

**Table 11:** Concentration of short chain fatty acids (SCFAs) in feces (mg/g dried feces).

Parameter (mg/g)	Group (Mean ± S.D.)				
	Normal	Hyperlipidemia Rats			
		Control	Simvastatin	CDF	Cellulose
Total SCFA	3.14 ± 0.58 ^a^	2.71 ± 1.02 ^ab^	2.37 ± 0.72 ^ab^	4.03 ± 0.76 ^a^	0.85 ± 0.58 ^b^
Acetic acid	1.88 ± 1.0 ^a^	1.28 ± 1.08 ^ab^	0.92 ± 0.65 ^b^	1.95 ± 1.03 ^a^	0.44 ± 0.54 ^b^
Propionic acid	0.72 ± 0.17 ^ab^	0.82 ± 0.67 ^ab^	0.78 ± 0.47 ^ab^	1.42 ± 0.57 ^a^	0.24 ± 0.38 ^b^
Butyric acid	0.54 ± 0.12 ^ab^	0.61 ± 0.33 ^a^	0.67 ± 0.39 ^a^	0.66 ± 0.09 ^a^	0.18 ± 0.28 ^b^

Means with different superscripted letters in the same row differ significantly (*p* < 0.05).

## Data Availability

The data used to support the findings of this study can be made available by the corresponding author upon request.

## References

[1] Sriroth K., Chollakup R., Chotineeranat S., Piyachomkwan K., Oates C.G. (2000). Processing of cassava waste for improved biomass utilization. Bioresour. Technol..

[2] Thailand Topioca Starch (2010). Treatment and Utilization of Root, Stem, and Pulp, in Treatment and Utilization of Wastewater and Solid Wastes. https://sustainablecassava.org/information-hub/cassava-value-chain/waste-treatment/.

[3] Wandee Y., Uttapap D., Puncha-arnon S., Puttanlek C., Rungsardthong V., Wetprasit N. (2014). Enrichment of rice noodles with fibre-rich fractions derived from cassava pulp and pomelo peel. Int. J. Food Sci. Technol..

[4] IUPAC (2006). Acute Toxicity in the Gold Book.

[5] Marques F.Z., Nelson E.M., Chu P., Horlock D., Fiedler A., Ziemann M., Tan J.K., Kuruppu S., Rajapakse N.W., El-osta A. (2017). High-fiber diet and acetated supplementation change the gut microbiota and prevent the development of hypertension and heart failure in hypertensive mice. Circulation.

[6] Mitamura R., Hara H., Aoyama Y., Takahashi T., Furuta H. (2003). Ingestion of water-soluble soybean fiber prevents osteopenia and hypercholesterolemia induced by ovariectomy in rats. J. Agric. Food Chem..

[7] Dabour N., El-Saadany K., Shoukry E., Hamdy S., Taïbi A., Kheadr E. (2022). The ability of yoghurt supplemented with dietary fibers or brans eatracted from wheat or rice to reduce serum lipids and enhance liver function in male hypercholesterolemic rats. J. Food Biochem..

[8] Anderson J., Deakins D.A., Floore T.L., Smith B.H., Whitis S.E. (1990). Dietary fiber and coronary heart disease. Crit. Rev. Food. Sci. Nutr..

[9] Esmael O., Sonbul S., Kumosani T.A., Mosalhy S.S. (2013). Hypolipiddemic effect of fruit fibers in rats fed with high dietary fat. Toxicol. Ind. Health.

[10] El-Sayed M. (2013). Effect of feeding on some dietary fibers for reducing blood cholesterol. J. Food Diary Sci..

[11] Kachenpukdee N., Santerre C., Ferruzzi M., Oonsivilai R. (2016). Enzymatic digestion optimization of dietary fiber from cassava pulp and their effect on mercury bioaccessibility and intestinal uptake from fish using an in vitro digestion/Caco-2 model. Int. Food Res. J..

[12] OECD (2022). OECD Guideline for Testing of Chemical: Acute Oral Toxicity-Acute Toxicity Class Method.

[13] Folch J., Lees M., Stanley G.S. (1957). A Simple Method for the Isolation and Purification of Total Lipids from Animal Tissues. J. Biol. Chem..

[14] Searcy R.L., Bergquist L.M. (1960). A new color reaction for the quantitation of serum cholesterol. Clin. Chim. Acta.

[15] de Almeida Jackix E., Monteiro E.B., Raposo H.F., Amaya-Farfán J. (2013). Cholesterol reducing and bile-acid binding properties of taioba (Xanthosoma sagittifolium) leaf in rats fed a high-fat diet. Food Res. Int..

[16] Henningsson Å., Björck I., Nyman M. (2001). Short-chain fatty acid formation at fermentation of indigestible carbohydrates. Food Nutr. Res..

[17] Sembries S., Dongowski G., Mehrländer K., Will F., Dietrich H. (2006). Physiological effects of extraction juices from apple, grape, and red beet pomaces in rats. J. Agric. Food Chem..

[18] Wong J.M., De Souza R., Kendall C.W., Emam A., Jenkins D.J. (2006). Colonic health: Fermentation and short chain fatty acids. J. Clin. Gastroenterol..

[19] Ali D., Soewarno N., Sumarno P.D., Sumaryo W. (2011). Cassava pulp as a biofuel feedstock of an enzymatic hydrolysis process. Makara J. Technol..

[20] Kosoom W., Charoenwattanasakun N., Ruangpanit Y., Rattanatabtimtong S., Attamangkune S. Physical, chemical and biological properties of cassava pulp. Proceedings of the 47th Kasetsart University Annual Conference, Kasesart.

[21] Rattanachomsri U., Tanapongpipat S., Eurwilaichitr L., Champreda V. (2009). Simultaneous non-thermal saccharification of cassava pulp by multi-enzyme activity and ethanol fermentation by Candida tropicalis. J. Biosci. Bioeng..

[22] Suksombat W., Lounglawan P., Noosen P. (2006). Energy and protein evaluation of five feedstuffs used in diet in which cassava pulp as main energy source for lactating dairy cows. Suranaree J. Sci. Technol..

[23] Jacometti G.A., Mello L.R., Nascimento P.H., Sueiro A.C., Yamashita F., Mali S. (2015). The physicochemical properties of fibrous residues from the agro industry. LWT-Food Sci. Technol..

[24] Pandey A., Soccol C.R., Nigam P., Soccol V.T., Vandenberghe L.P., Mohan R. (2000). Biotechnological potential of agro-industrial residues. II: Cassava bagasse. Bioresour. Technol..

[25] Lacourse N.L., Chicalo K., Zallie J.P., Altieri P.A. (1994). Dietary Fiber Derived from Tapioca and Process Therefor. U.S. Patent.

[26] Hong Y., Zi-jun W., Jian X., Ying-jie D., Fang M. (2012). Development of the dietary fiber functional food and studies on its toxicological and physiologic properties. Food Chem. Toxicol..

[27] Gao Y., Shen J., Yin J., Li C., Fu C., Cho S. (2013). A subchronic dietary toxicity study of rice hull fiber in rats. Food Chem. Toxicol..

[28] Uberoi S., Vadhera S., Soni G. (1992). Role of dietary fiber from pulses and cereals as hypocholesterolemic and hypolipidemic agent. J. Food Sci. Technol..

[29] Fang Y., Ma J., Lei P., Wang L., Qu J., Zhao J., Liu F., Yan X., Wu W., Jin L. (2023). Konjac Glucomannan: An Emerging Specialty Medical Food to Aid in the Treatment of Type 2 Diabetes Mellitus. Foods.

[30] Chirinang P., Oonsivilai R. (2018). Physicochemical properties, in-vitro binding capacities for lard, cholesterol, bile acids and assessment of prebiotic potential of dietary fiber from cassava pulp. Int. Food Res. J..

